# Nanorod-Shaped Basic Al_2_O_3_ Catalyzed *N,N*-Diformylation of Bisuracil Derivatives: A Greener “NOSE” Approach

**DOI:** 10.1155/2013/793159

**Published:** 2013-06-26

**Authors:** Vijay K. Das, Ashim J. Thakur

**Affiliations:** Department of Chemical Sciences, Tezpur University (A Central University), Napaam, Assam 784028, India

## Abstract

A feasible “NOSE” (nanoparticles-catalyzed organic synthesis enhancement) protocol has been developed for *N,N*-diformylation of bisuracil derivatives using nano-Al_2_O_3_ rods as an efficient, inexpensive, and recyclable catalyst under solvent-free reaction condition at 40°C. The catalyst was reused up to the 4th cycle without affecting the rate and yield of the *N,N*-diformylation products appreciably.

## 1. Introduction

The exercise of metal/metal oxide nanoparticles as a frontier between the homogeneous catalysis and heterogeneous catalysis [1] in organic synthesis has invoked tremendous interests [2] in the recent times. The interesting features inherited with these small particle sizes are their large surface area along with more edges and corners and distinct electronic, optical, magnetic, thermal, and chemical properties [3–5]. The crucial role of nanoparticles in organic transformations is their excellent catalytic activity, straightforward recoverability, better selectivity, criteria of evolution, and their versatile role in green chemistry [6–10]. Thus, the domain of metal nanoparticle catalysis [11–13] should offer opportunities for mining new chemical reactions [14–16] which include the synthesis of biologically important and synthetically challenging natural products. In the context of green chemistry [17], organic synthesis in solvent-free reaction condition [18–21] has occupied a significant position in the recent years since solvent-free reaction condition involves the best reaction medium with “no medium” [22]. 

 One of the key motifs present in the biopolymer RNA [23–26] is uracil, a nucleobase of the pyrimidine family which participates in various functions in our life processes [27]. Uracil derivatives also have several potent medicinal properties such as bronchodilators and anticancer [28, 29], antiallergic [30, 31], antiviral [32, 33], antihypertensive, and adenosine receptor antagonists [34, 35]. Recently, our research group reported a greener protocol for the synthesis of bisuracil derivatives [36]. Bisuracil and their analogues have also been isolated from marine sea hare *Dolabella auricularia *[37]. Some of the *N*-substituted bisuracil analogues have been screened for bioactivities against several diseases [38].

**Scheme 1:**
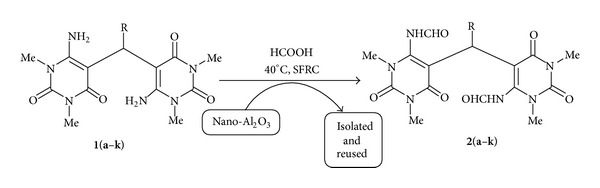
*N,N*-diformylation of bisuracil derivatives **1**(**a–k**).

To explore the possible applications of the metal/metal oxide nanoparticles in organic synthesis, we have been focusing on the advancement of a protocol termed “NOSE” (nanoparticles-catalyzed organic synthesis enhancement) [39–41] chemistry in our laboratory. To the best of our knowledge, there has been no report on nano-rod-shaped Al_2_O_3_ catalyzed *N*,*N*-diformylation of bisuracil derivatives. Recently, we reported *N*-formylation of amines catalyzed by nano-Al_2_O_3_ under solvent-free reaction condition [39]. This work inspired us to focus on nano-Al_2_O_3_ catalysis for the *N*,*N*-diformylation of bisuracil analogous. Therefore, in this paper, we wish to account for the same (Scheme 1).

Nano-Al_2_O_3_ draws our attention due to its crystalline size and shape, abrasive and insulating properties, less toxicity, large surface area, basic surface characteristics, high resistant towards bases and acids and excellent wear resistance [40–44].

## 2. Materials and Methods

### 2.1. General Experimental Methods

Rod-shaped nano-Al_2_O_3_ (the average particle diameter is 8.12 nm and average length 25.5 nm, *S*_BET_ = 185.63 m^2^ g^−1^, *ρ* = 3.98 g cm^−3^, and purity is 99.99%) were purchased from Sigma Aldrich and used as received. The chemicals and reagents were purchased from Sigma-Aldrich, Merck, M/S S.D. Fine Chemicals Pvt. Ltd., and Loba Chemie, and used without further purification. The XRD pattern was recorded with Rigaku X-ray diffractometer. Melting points were determined in a Büchi 504 apparatus. IR spectra were recorded as KBr pallets in a Nicolet (Impact 410) FT-IR spectrophotometer. ^1^H and ^13^C NMR spectra were recorded in a 400 MHz NMR spectrophotometer (JEOL, JNM ECS) using tetramethylsilane (TMS) as the internal standard, and coupling constants are expressed in Hertz. Elemental analyses were carried out in a Perkin-Elmer CHN analyser (2400 series II). Mass spectra were recorded with a Waters Q-TOF Premier and an Acquity UPLC spectrometer. Visualization was accomplished with UV lamp or I_2_ stain. Reactions were monitored by thin-layer chromatography using aluminium sheets with silica gel 60 F_254_ (Merck).

### 2.2. General Procedure for *N,N*-Diformylation of Bisuracil Derivatives

**Scheme 2:**
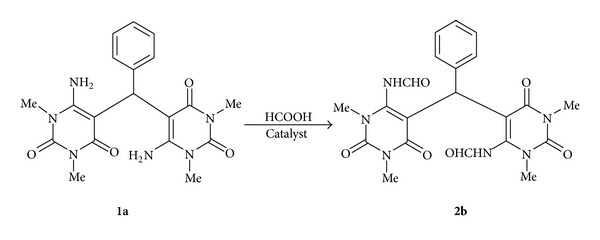
Optimization of reaction condition.

In a two-neck round bottom flask (50 mL), nanorod-shaped basic Al_2_O_3_ (7.0 mol%, 7.12 mg) were taken, and then **1g** (1.0 mmol, 414 mg) and formic acid (98%, 6.0 mmol, 0.23 mL) were added. After that, it was allowed to stir on a pre heated oil bath at 40°C for the required time (the progress of the reaction was judged by TLC). The reaction mixture was brought to room temperature after its completion, and ethyl acetate (3 × 10 mL) was added and then centrifuged (3,000 r.p.m) to recover the nanocatalyst. Having done this, the reaction mixture was washed with water and brine, dried over anhydrous Na_2_SO_4_, and concentrated in a rotary evaporator, and finally the crude product was purified by column chromatography (30% ethyl acetate: hexane as an eluent). The recovered catalyst was washed with hot ethanol (3 × 10 mL) to remove the organic impurities, decanted, dried in an oven at 80°C for 6 h, and reused for evaluating the performance in the next run in the reaction as shown in Scheme 2.

## 3. Results and Discussion

With the previously reported catalyst characterizations in hand [39], to begin with, reaction of 6,6′-diamino-1,1′,3,3′-tetramethyl-5,5′-(benzylidene)bis[pyrimidine-2,4 (1H, 3H)-dione] [36] (**1a**, 1 mmol) with formic acid (6 mmol) was chosen as the model reaction (Scheme 2).

The optimization of the various parameters of this reaction is elaborated in Table 1. Initially, the reaction was carried out without using catalyst under solvent-free reaction condition at 40°C and 80°C which did not yield any product (Table 1, entries 1 and 2). Various solvents were also tested under the mentioned condition, but they all failed (Table 1, entries 3–11) to provide any product. These negative results suggested that we look for an effective catalyst in the present study. Next, various Lewis acid-base catalysts (Table 1, entries 12–14) along with the nanocatalysts (Table 1, entries 15–18) were surveyed to observe the influence on rate and yield of *N,N*-diformylation of **1a** which were not fruitful. Interestingly, nanorod-shaped basic Al_2_O_3_ stood out as a choice of catalyst at 7 mol% loading (Table 1, entry 15) under solvent-free reaction condition at 40°C. During the course of our experiment, we observed that at higher temperature (Table 1, entry 19) and at lower/higher catalyst loading the yield of the products was poor (Table 1, entries 20–22). Thus, the yield of *N,N*-diformylation product of bisuracil derivatives is highly dependent upon the temperature and catalyst loading. 

With this supportive optimized reaction condition in hand, a series of bisuracil derivatives (entries 1–11) bearing different aliphatic, aromatic, and heterocyclic moieties were examined to explore the scope and limitations of this reaction and the outcomes are presented in Table 2. It is clear from Table 2 that bisuracil derivatives carrying both electron donating and electron withdrawing groups in benzene ring underwent *N,N*-diformylation reaction smoothly producing good yields (Table 2, entries 1–8). However, longer reaction time was required for bisuracil derivatives substituted with furan and alkyl groups (Table 2, entries 9–11). It is worth mentioning that 6-amino-1,3-dimethyluracil when treated with formic acid under the current condition gave *N,N*-diformylation product in lower yield (26%, 9 h). The reactions were found to be clean, and no side products were formed.

 To test the recyclability (vide Scheme 2) of nano-Al_2_O_3_, it was separated from the reaction mixture by adding ethyl acetate (10 mL), centrifuged at 3,000 rpm, to pellet out the catalyst. The separated particles were washed with hot ethanol (3 × 10 mL) to remove the organic impurities, decanted, dried in an oven at 80°C for 6 h, and reused for further reactions. The efficiency of the catalyst was found to be unaffected up to 4th run, and after that, its action started to decrease as shown in Table 3. The TONs were also retained from fresh up to the 5th cycle, and after that it decreased considerably.

**Figure 1:**
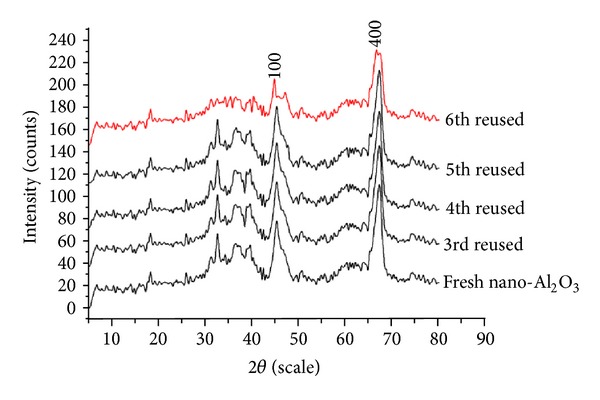
Comparison of XRD of fresh nano-Al_2_O_3_ with the recovered ones.

 The recovered catalyst was also investigated through powder XRD and it was compared with the fresh nano-Al_2_O_3_ (Figure 1). In the powder XRD of the recovered catalyst after 6th run (Figure 1), the intensity of the peaks (4 0 0) and (1 0 0) weakened and became broad. It might be due to the blockage of the pores of the catalyst which caused a decrease in effective active sites and also due to the dislocation of the crystal planes after each run which in turn decreased the yield.

**Figure 2:**
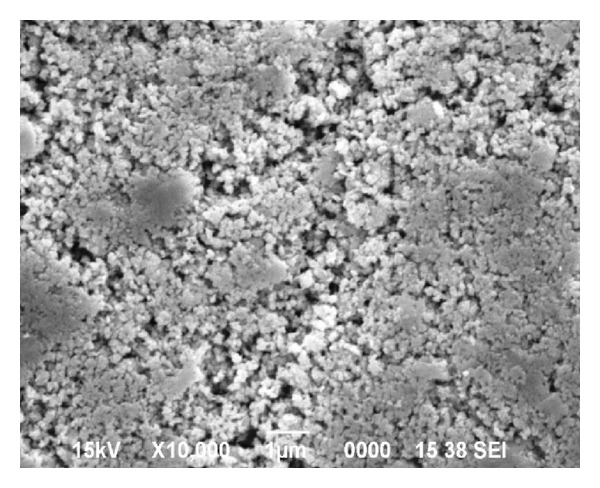
SEM image of recovered nano-Al_2_O_3_ after 4th run.

The SEM micrograph of the fresh nano-Al_2_O_3_ previously reported by us [39] was also compared with the recycled one (Figure 2) under the present study. As indicated in Figure 2, the recycled nano-Al_2_O_3_ revealed the aggregation of the particles responsible for reducing its surface area and hence deactivated the catalyst after 4th run which caused the lower yield of product. 

## 4. Conclusions

In conclusion, we have demonstrated a novel method for synthesis the *N*,*N*-diformylation of bisuracil derivatives in good yield under solvent-free reaction condition at 40°C catalyzed by recyclable nano-Al_2_O_3_ rods. Nano-Al_2_O_3_ catalyzed organic transformations are less explored. We believe that this work would find wide applications for new chemical transformations, including those which enable the synthesis of complex natural products and derivatives.

## Supplementary Material

Physical and Spectroscopic Data of compounds (2a-2k):
The physical and spectroscopic data of the synthesized *N,N*-diformylation products (2a-2k)

## Figures and Tables

**Table 1:** Optimization of the reaction conditions for the *N,N*-diformylation of **1a** (Scheme 1).

Entry	Catalyst	Solvent	Temp. (°C)	Time (h)	Yield (%)^b^
1	None	Solvent-free	40	9	NR^c^
2	None	Solvent-free	80	9	NR^c^
3	None	H_2_O	40	12	NR^c^
4	None	CH_3_CN	40	12	NR^c^
5	None	MeOH	40	12	NR^c^
6	None	EtOH	40	12	NR^c^
7	None	THF	40	12	NR^c^
8	None	Toluene	40	12	NR^c^
9	None	DMSO	40	12	NR^c^
10	None	Xylene	40	12	NR^c^
11	None	DMF	40	12	NR^c^
12^d^	K_2_CO_3_	Solvent-free	40	12	NR^c^
13^d^	PPh_3_	Solvent-free	40	12	NR^c^
14^d^	Imidazole	Solvent-free	40	10	Trace
15^d^	Nano-Al_2_O_3_^i^	Solvent-free	40	45 min	70
16^d^	Nano-MgO^j^	Solvent-free	40	3	34
17^d^	Nano-Fe_2_O_3_^k^	Solvent-free	40	5	12
18^d^	Nano-TiO_2_^l^	Solvent-free	40	4	8
19^d^	Nano-Al_2_O_3_^i^	Solvent-free	80	2	43
20^e^	Nano-Al_2_O_3_^i^	Solvent-free	40	3	25
21^f^	Nano-Al_2_O_3_^i^	Solvent-free	40	4	17
22^g^	Nano-Al_2_O_3_^i^	Solvent-free	40	6	8

^a^Reaction conditions: bisuracil **1a** (1 mmol, 0.454 g), formic acid (6 mmol, 0.66 mL), and solvent (5 mL). ^b^Isolated yields. ^c^No reaction was observed. ^d^7 mol% catalyst was used. ^e^5 mol% catalyst was used. ^f^3 mol% catalyst was used. ^g^10 mol% catalyst was used. ^h^1 mol% catalyst was used. ^i^Particles size (17.4–16.4 nm). ^j^Particles size (<50 nm). ^k^Particles size (12 nm). ^l^Particles size (<80 nm).

**Table 2:** Nano-Al_2_O_3_ catalyzed *N,N*-diformylation of uracil and bisuracil derivatives.

Entry	“R” in **1**	Product **2**	Time (min)	Yield (%)^a,b^
1	C_6_H_5_(**1a**)	**2a**	45	70
2	*p*-OMeC_6_H_4_ (**1b**)	**2b**	60	68
3	*p*-ClC_6_H_4_ (**1c**)	**2c**	75	58
4	*p*-OHC_6_H_4_ (**1d**)	**2d**	90	55
5	*p*-NO_2_C_6_H_4_ (**1e**)	**2e**	90	52
6	*p*-MeC_6_H_4_ (**1f**)	**2f**	70	60
7	*o*-OHC_6_H_4_ (**1g**)	**2g**	100	52
8	*m*-NO_2_C_6_H_4_ (**1h**)	**2h**	90	65
9	CH_3_ (**1i**)	**2i**	100	52
10	CH_3_(CH_2_)_3_(**1j**)	**2j**	120	44
11	2-furyl (**1k**)	**2k**	150	57

^a^6 mmol of formic acid was used. ^b^Isolated yield. ^c^Products were characterized by IR and NMR (^1^H and ^13^C) spectroscopy, MS, and also melting points.

**Table 3:** Recycling study of nano-Al_2_O_3_.

Entry	No. of cycles	Time (min)	Yield (%)^b^	TONs
1	Fresh	45	70	88
2	1st run	45	70	88
3	2nd run	45	70	88
4	3rd run	45	70	88
5	4th run	45	70	88
6	5th run	60	58	76
7	6th run	180	40	70

^a^Reaction conditions: 2 mmol of **2b**, 12 mmol formic acid, and 7 mol% basic nano-Al_2_O_3_, 40°C. ^b^Yields refer to the isolated pure products.
