# Supplementary material for: Nanorod-Shaped Basic Al2O3 Catalyzed N,N-Diformylation of Bisuracil Derivatives: A Greener “NOSE” Approach
Source: ISRN Org Chem. 2013 Jun 26;2013:793159. doi: 10.1155/2013/793159 (PMC3767338; doi:10.1155/2013/793159)
Supplement: Supplementary file 1 — Physical and Spectroscopic Data of compounds (2a-2k): The physical and spectroscopic data of the synthesized N,N-diformylation products (2a-2k) [file 793159.f1.doc]

**Supporting information**

**Nano-rod shaped basic Al_2_O_3_ catalyzed *N,N*-diformylation of bis-uracil derivatives: A greener ‘NOSE’ approach**

Vijay Kumar Das and Ashim Jyoti Thakur*

Department of Chemical Sciences,

Tezpur University (A Central University),

Tezpur, Napaam 784028, Assam, India,

Fax: +91 (3712) 267005/6,

E-mail: ashim@tezu.ernet.in

**Physical and Spectroscopic Data of compounds** (**2a-2k**)**:**

The physical and spectroscopic data of the synthesized *N,N*-diformylation products (2a-2k) are given below:

**6,6'-Diformamide-1,1',3,3'-tetramethyl-5,5'-(benzylidene)bis[pyrimidine-2,4(1H,3H)-dione] (Table 2, entry 2a)**:

A pale yellow powder; R_f_ = 0.42 (30% AcOEt:hexane); mp 252-254 ºC; ^1^H NMR (400 MHz, CDCl_3_, TMS): δ 3.19 (s, 3H, NCH_3_), 3.31 (s, 3H, NCH_3_), 3.47 (s, 3H, NCH_3_), 3.54 (s, 3H, NCH_3_), 4.95 (s, 1H, CH), 6.56 (br, s, 2H), 7.11-7.27 (m, 5 H, Ar-H), 8.67 (s, 2H, CHO); ^13^C NMR (100 MHz, CDCl_3_, TMS): δ 27.8, 28.1, 28.8, 28.9, 35.4, 87.1, 88.3, 125.2, 126.3, 127.2, 137.5, 150.4, 153.1, 154.2, 162.8, 164.2, 170.1, 170.8; IR (KBr pellets) ν_max_ (cm^-1^): 3450 (NH), 2830 (CH), 2810 (CH), 1710 (CO), 1680 (CO); m/z 454.16 [M^+^]; Anal. Calcd (%) for C_21_H_22_N_6_O_7_: C, 55.50; H, 4.88; N, 18.49. Found C, 55.10, H, 4.48, N, 18.09.

**6,6'-Diformamide-1,1',3,3'-tetramethyl-5,5'-(4-methoxybenzylidene)bis-[pyrimidine-2,4 (1H,3H)-dione] (Table 2, entry 2b)**:

White solid; R_f_ = 0.34 (30% AcOEt:hexane); mp 243–245 ºC; ^1^H NMR (400 MHz, CDCl_3_,

TMS): δ 3.21 (s, 3H, NCH_3_), 3.32 (s, 3H, NCH_3_), 3.39 (s, 3H, NCH_3_), 3.43 (s, 3H, NCH_3_), 3.49 (s, 3H, OCH_3_), 4.48 (s, 1H, CH), 6.51 (br, s, 2H, NH), 7.18 (d, *J* = 8.12 Hz, 2H, Ar-H), 7.21 (d, *J* = 8.12 Hz, 2H, ArH), 9.51 (s, 1H, CHO), 9.13 (s, 1H, CHO); ^13^C NMR (100 MHz, CDCl_3_, TMS): δ 29.1, 29.4, 29.6, 29.8, 32.8, 58.6, 87.1, 88.7, 114.2, 128.1, 130.3, 152.1, 154.6, 157.7, 158.7, 164.5, 166.1, 170.7; IR (KBr pellets) ν_max_ (cm^-1^): 3394 (NH), 2953 (CH), 2822 (CH), 1725 (CO), 1679 (CO); m/z 484.17 [M^+^]; Anal. Calcd (%) for C_22_H_24_N_6_O_7_: C, 54.54; H, 4.99; N, 17.35. Found C, 54.12, H, 4.59, N, 16.95.

**6,6'-Diformamide-1,1',3,3'-tetramethyl-5,5'-(4-chlorobenzylidene)bis-[pyrimidine-2,4 (1H, 3H)-dione] (Table 2, entry 2c)**:

Off white solid; R_f_ = 0.38 (30% AcOEt:hexane); mp 185–188 ºC; ^1^H NMR (400 MHz, DMSO, TMS): δ 3.25 (s, 3H, NCH_3_), 3.31 (s, 3H, NCH_3_), 3.48 (s, 6H, NCH_3_), 5.68 (s, 1H, CH), 7.48-7.68 (m, 4H, Ar-H), 8.35 (s, 2H, CHO); ^13^C NMR (100 MHz, DMSO, TMS): δ 29.1, 29.3, 29.5, 29.7, 36.5, 88.3, 88.7, 127.8, 128.3, 131.5,136.7, 151.2, 153.8, 155.4, 164.8, 165.7, 171.1; IR (KBr pellets) ν_max_ (cm^-1^): 3376 (NH), 2948 (CH), 2834 (CH), 1732 (CO), 1687 (CO); m/z 488.12 [M^+^]; Anal. Calcd (%) for C_21_H_21_ClN_6_O_6_: C, 51.59; H, 4.33; N, 17.19. Found C, 51.19, H, 3.93, N, 16.79.

**6,6'-Diformamide-1,1',3,3'-tetramethyl-5,5'-(4-hydroxybenzylidene)bis-[pyrimidine-2,4 (1H, 3H)-dione] (Table 2, entry 2d)**:

Light brown solid; R_f_ = 0.24 (30% AcOEt:hexane); mp 217-218 ºC; ^1^H NMR (400 MHz, DMSO, TMS): δ 2.97 (s, 3H, NCH_3_), 3.03 (s, 3H, NCH_3_), 3.07 (s, 3H, NCH_3_), 3.22 (s, 3H, NCH_3_), 4.88 (s, 1H, CH), 6.72 (br, 2H, NH), 7.81-8.02 (m, 4H, Ar-H), 8.87 (d, *J* = 7.52 Hz, 2H, CHO) 10.21 (br, 1H, OH); ^13^C NMR (100 MHz, DMSO, TMS): δ 29.4, 29.6,34.8,114.7, 127.3, 127.7, 129.6, 151.7, 155.6, 168.9, 169.2, 170.6; IR (KBr pellets) ν_max_ (cm^-1^): 3398 (NH), 1705 (CO), 2957 (CH), 2827 (CH), 1655 (CO); m/z 470.15 [M^+^]; Anal. Calcd (%) for C_21_H_22_N_6_O_7_: C, 53.62; H, 4.71; N, 17.86. Found C, 53.22, H, 4.31, N, 17.46.

**6,6'-Diformamide-1,1',3,3'-tetramethyl-5,5'-(4-nitrobenzylidene)bis-[pyrimidine-2,4(1H, 3H)-dione] (Table 2, entry 2e)**:

Light brown solid; R_f_ = 0.29 (30% AcOEt:hexane); mp 205–208 ºC; ^1^H NMR (400 MHz, CDCl_3_, TMS): δ 3.33 (s, 3H, NCH_3_), 3.41 (s, 3H, NCH_3_), 3.43 (s, 3H, NCH_3_), 3.51 (s, 3H, NCH_3_), 6.61 (br, s, 2H, NH), 8.02-8.31 (m, 4H, Ar-H), 9.45 (s, 2H, CHO); ^13^C NMR (100 MHz, CDCl_3_, TMS): δ 28.4, 29.5, 29.8, 30.2, 36.7, 87.6, 88.7, 122.8, 128.3, 141.5, 149.7, 152.1, 154.3, 156.3, 165.1, 167.2, 173.3, 173.7; IR (KBr pellets) ν_max_ (cm^-1^): 3433 (NH), 2933 (CH), 2818 (CH), 1728 (CO), 1678 (CO); m/z 499.15 [M^+^]; Anal. Calcd (%) for C_21_H_21_N_7_O_8_: C, 50.50; H, 4.24; N, 19.63. Found C, 50.10, H, 3.84, N, 19.43.

**6,6'-Diformamide-1,1',3,3'-tetramethyl-5,5'-(4-methylbenzylidene)bis-[pyrimidine-2,4(1H,3H)-dione]** **(Table 2, entry 2f)**:

White solid; R_f_ = 0.56 (30% AcOEt:hexane); mp 239–242 ºC; ^1^H NMR (400 MHz, CDCl_3_, TMS): δ 2.23 (s, 3H, CH_3_), 2.38 (s, 3H, NCH_3_), 2.77 (s, 3H, NCH_3_), 2.94 (s, 3H, NCH_3_), 3.19 (s, 3H, NCH_3_), 5.19 (s, 1H, CH), 6.39 (br, s, 2H, NH), 7.34 (d, 2H, *J* = 8.35 Hz, Ar-H), 7.57 (d, 2H, *J* = 8.35 Hz, Ar-H), 8.87 (s, 2H, CHO); ^13^C NMR (100 MHz, CDCl_3_, TMS): δ 19.8, 27.3, 27.7, 29.1, 29.6, 35.8, 87.7, 88.5, 127.4, 129.0, 135.5, 136.8, 151.1, 153.4, 154.8, 162.8, 163.6, 169.1, 169.8; IR (KBr pellets) ν_max_ (cm^-1^): 3441 (NH), 2928 (CH), 2870 (CH), 1713 (CO), 1632 (CO); m/z 468.18 [M^+^]; Anal. Calcd (%) for C_22_H_24_N_6_O_6_: C, 56.40; H, 5.16; N, 17.94. Found C, 56.10, H, 4.76, N, 17.54.

**6,6'-Diformamide-1,1',3,3'-tetramethyl-5,5'-(2-hydroxybenzylidene)bis-[pyrimidine-2,4 (1H,3H)-dione] (Table 2, entry 2g)**:

off white powder; R_f_ = 0.35 (30% AcOEt:hexane); mp 210-214 ºC; ^1^H NMR (400 MHz, CDCl_3_, TMS): δ 3.11 (s, 3H, NCH_3_), 3.35 (s, 3H, NCH_3_), 3.50 (s, 3H, NCH_3_), 3.59 (s, 3H, NCH_3_), 4.85 (s, 1H, CH), 5.77 (s, 1H, OH), 6.39 (br, s, 2H, NH), 7.10-7.26 (m, 5H, Ar-H), 8.59 (s, 2H, CHO); ^13^C NMR (100 MHz, CDCl_3_, TMS): δ 27.6, 28.3, 29.3, 29.4, 30.1, 87.9, 93.4, 115.7, 123.5, 125.3, 128.0, 128.4, 150.5, 150.6, 151.4, 151.6, 154.4, 161.3, 164.2, 168.9; IR (KBr pellets) ν_max_ (cm^-1^): 3430 (NH), 2830 (CH), 2812 (CH), 1710 (CO), 1680 (CO); m/z 470.15 [M^+^]; Anal. Calcd (%) for C_21_H_22_N_6_O_7_: C, 53.62; H, 4.71; N, 17.86. Found C, 53.22, H, 4.31, N, 17.46.

**6,6'-Diformamide-1,1',3,3'-tetramethyl-5,5'-(3-nitrobenzylidene)bis-[pyrimidine-2,4 (1H,**

**3H)-dione]** (**Table 2, entry 2h)**:

Pale brown solid; R_f_ = 0.30 (30% AcOEt:hexane); mp 197-198 ºC; ^1^H NMR (400 MHz, CDCl_3_, TMS): δ 3.37 (s, 3H, NCH_3_), 3.50 (s, 3H, NCH_3_), 3.52 (s, 3H, NCH_3_), 3.55 (s, 3H, NCH_3_), 4.88 (s, 1H, CH), 6.68 (br, s, 2H, NH), 7.37-7.83 (m, 4H, Ar-H), 8.35 (s, 2H, CHO); ^13^C NMR (100 MHz, CDCl_3_, TMS): δ 28.8, 29.3, 29.8, 30.2, 35.6, 85.8, 87.3, 121.5, 122.3, 129.3, 134.2, 142.5, 148.8, 152.3, 154.3, 154.7, 164.7, 166.3, 169.3, 170.1; IR (KBr pellets) ν_max_ (cm^-1^): 3414 (NH), 3095 (CH), 2902 (CH), 1713 (CO), 1696 (CO); m/z 499.15 [M^+^]; Anal. Calcd (%) for C_21_H_21_N_7_O_8_: C, 50.50; H, 4.24; N, 19.63. Found 50.10, H, 3.84, N, 19.23.

**6,6'-Diformamide-1,1',3,3'-tetramethyl-5,5'-(ethylidene)bis-[pyrimidine-2,4(1H, 3H)-dione]** **(Table 2, entry 2i)**:

White solid; R_f_ = 0.64 (30% AcOEt:hexane); mp 288–290 ºC; ^1^H NMR (400 MHz, CDCl_3_,

TMS): δ 1.28 (d, *J* = 6.83 Hz, 3H, CH_3_), 2.71 (s, 3H, NCH_3_), 2.86 (s, 3H, NCH_3_), 2.88 (s, 3H, NCH_3_), 2.92 (s, 3H, NCH_3_), 3.77-4.13 (m, 1H, CH), 6.01 (br, s, 2H, NH), 8.15 (s, 2H, CHO); ^13^C NMR (100 MHz, CDCl_3_, TMS): δ 12.4, 21.3, 28.1, 28.3, 29.3, 29.9, 88.5, 89.1, 148.0, 149.2, 152.2, 159.6, 161.3, 165.3, 167.6; IR (KBr pellets) ν_max_ (cm^-1^): 3409 (NH), 3041 (CH), 2989 (CH), 1705 (CO), 1688 (CO); m/z 392.14 [M^+^]; Anal. Calcd (%) for C_16_H_20_N_6_O_6_: C, 48.98; H, 5.14; N, 21.42. Found 48.58, H, 4.74, N, 21.02.

**6,6'-Diformamide-1,1',3,3'-tetramethyl-5,5'-(pentylidene)bis-[pyrimidine-2,4 (1H, 3H)-dione)** **(Table 2, entry 2j)**:

White solid; R_f_ = 0.68 (30% AcOEt:hexane); mp 108–111 ºC; ^1^H NMR (400 MHz, CDCl_3_,

TMS): δ 0.98-1.02 (m, 3H, CH_3_), 1.38-1.42 (m, 4H, CH_2_CH_2_), 2.38-2.51 (m, 2H, CH_2_), 3.37 (s, 3H, NCH_3_), 3.42 (s, 3H, NCH_3_), 3.47 (s, 3H, NCH_3_), 3.49 (s, 3H, NCH_3_), 4.25-4.31 (m, 1H, CH), 6.69 (br, 2H, NH), 8.82 (s, 2H, CHO); ^13^C NMR (100 MHz, CDCl_3_, TMS): δ 14.1, 22.7, 26.5, 27.1, 28.8, 29.2, 29.8, 30.8, 35.2, 88.7, 89.6, 151.2, 151.8, 152.3, 154.4, 164.4, 165.6, 170.7, 171.0; ν_max_ (cm^-1^): 3455 (NH), 3012 (CH), 2882 (CH), 1722 (CO), 1693 (CO); m/z 434.19 [M^+^]; Anal. Calcd (%) for C_19_H_26_N_6_O_6_: C, 52.53; H, 6.03; N, 19.34. Found 52.13, H, 5.63, N, 18.94.

**6,6'-Diformamide-1,1',3,3'-tetramethyl-5,5'-(2-furyl)bis-[pyrimidine-2,4(1H,3H)-dione]** **(Table 2, entry 2k)**:

Yellow viscous oil; R_f_ = 0.12 (30% AcOEt:hexane); ^1^H NMR (400 MHz, CDCl_3_, TMS): δ 2.71

(s, 3H, NCH_3_), 2.75 (s, 3H, NCH_3_), 2.87 (s, 3H, NCH_3_), 2.91 (s, 3H, NCH_3_), 5.11 (s, 1H, CH), 5.88 (d, 1H, Furan-H), 6.18 (d, 1H, Furan-H), 6.27 (d, 1H, Furan-H), 6.55 (br, 2H, NH), 9.22 (s, 2H, CHO); ^13^C NMR (100 MHz, CDCl_3_, TMS): δ 28.3, 28.5, 29.1, 30.2, 87.1, 105.2, 110.5, 141.3, 151.2, 152.6, 153.6, 164.4, 170.3; ν_max_ (cm^-1^): 3348 (NH), 2974 (CH), 2862 (CH), 1996 (CO), 1636 (CO); m/z 444.14 [M^+^]; Anal. Calcd (%) for C_19_H_20_N_6_O_7_: C, 51.35; H, 4.54; N, 18.91. Found 50.95, H, 4.14, N, 18.51.
